# About Homeopathy

**Published:** 1873-07

**Authors:** P. H. Hayes


### ABOUT HOMEOPATHY.

BY P. H. HAYES, M. D.

The recent expulsion of several members of the Massachusetts Medical Society, for treating diseases Homeopathically, may fairly raise the enquiry as to what Homeopathy really is, and why it is so dishonorable to practice it, that such as do so, should be expelled from so ancient and honorable an organization as the Massachusetts Medical Society.

A physician of any grasp of mind and research into medical theory and practice, sees that there are, or may be, several ways of treating the same disease, involving different but sound principles of practice, and the question ever recurs, which is the best means involving the safest and surest principle in any particular or given case.

Thus a physician may treat a case of erysipelas with either cool or warm local applications, or with such local applications as iodine and nitrate of silver, and each involving a different principle, or he may treat a typhoid fever with the slow pulse remedies, aconite, gelsemiums, veratrum, or with tepid and salt sponging, or the wet sheet envelopment, or he may resort to frequent draughts of slightly saline waters, each involving a somewhat different principle, but all aiming at the same great result, the lowering of the pulse, and the bringing on of perspirations and the other secretions.

If, then, there be many principles and many methods of treatment and cure, he must be the wise man who dares adopt each and all as they may be needed, or always the best principle and method in all the circumstances.—And he must be the quack who knows and adopts only one idea and method, and tries to cure everything he may meet in this narrow way.

If Homeopathy be simply a method of using the secondary instead of the primary effects of medicine in curing diseases, it is certainly a safe and true method, but far from new, and does not need any such high sounding word as "Homeopathy" to express it.

The profession have known from immemorial time, that inflammations differ specifically in character. Thus common inflammation is unlike erysipelatous inflammation, and this again is specifically different from rheumatic inflammation, and this again from scrofulous inflammation. So the inflammation excited by nitrate of silver or iodine, differs from all others in certain characters. Now any medicine causing an inflammation may be used to cure an inflammation specifically different from itself. Thus I remember now a little girl whose ear and cheek near by were swollen with erysipelas. I immediately burnt the cheek slightly with caustic all around this line of erysipelas, and the erysipelatous inflammation never passed beyond this. The specific inflammation caused by the caustic kept the erysipelatous inflammation from moving any farther in that direction.

Now notice the principle involved; one inflammation cures another, *like* itself in many points, yet in a sense specifically *unlike* it.—The same principle is involved in the local application of nitrate of silver to the eye.—Here is a true *similia similibus.*

But this law has no wide extent of application. It is far from being the best method when applied to many internal or external diseases. There is another principle involved in this method of cure which is true, but also has only a limited range of application. And this is, that if the primary effect of any medicine be *like* the disease, the secondary is *unlike* it, and *if* unlike, tends to cure it. Again the primary effect is short, the secondary long and more permanent, and therefore this secondary effect tends to establish itself in the system, and being unlike the disease tends to cure it, yet this is not new, and the man who commits himself to this one idea must come under the ban of a large and liberal minded association of physicians. The greatest charlatanry the world knows is, I believe, covered up under what is known as Homeopathic practice.

I have heard one Homeopathist charge that another physician could not hold his patients long, but how did he, the Homeopath do the thing. So many drops of so many attenuations in so many tumblers of water, with special directions to take in alternation, or so many powders with the dose, and the times to be dropped upon the tongue. This method puts off patients, carries with it a show of science, saves the doctors much trouble, keeps the patient occupied, and satisfies the popular propensity for dosing. Thus are unimportant sensations continually prescribed for, thus are patients taught, and in the most direct way, to value these sensations, to magnify their sufferings, and watch for symptoms to tell the doctor. In this way is a sort of tyrrany established over the minds of patients, and the expectation of recovery sometimes for months together kept up in a purely artificial manner. I remember once asking one of this class of patients familiary, "how do you do to-day?" "Oh," she said," "I really don't know how I am, it keeps me so busy to watch for symptoms to tell the doctor." What tyrrany could be more subtle, refined and cruel than this, to set patients to watching and weighing their sensations that they may have somewhat to report to their doctor. Does not every physician of brains know that the subtle and exceedingly injurions influence of fear enters into all this study of self-sensations, and that sensitive patients are sure to bring to the doctor a new assortment of sensations every time they come to his office, or he visits them at their chambers. And is there any end to this thing?

The very laws and principles of refined Homeopathic practice tend to this very result of creating and perpetuating this sort of invalidism as certainly as though the system had been ingeniously contrived for no other purpose.

I believe many persons have in this way been made invalids for life, wretched, self-conscious and melancholy, in bondage to bodily sensations from which from long habit they seem unable to free themselves.

Mrs. E. began to tell me one morning about her eyes, and at that moment they began to ache and run water, as she soon after told me. Mrs. C. is shut up in a dark room just now with inflammation of the eyes, and, says she, the very *thought* of seeing with my eyes makes them feel hot and sends a flow of hot, scalding tears down my cheeks. What wise physician does not know that it is thus with all our sensations, the natural and the pleasureable as well as the morbid and the painful, that they are intensified by attention to them. The converse of this statement is also equally strikingly true; thus soldiers often did not know they were wounded until the battle was over and the attention called to the fact, and it is related of Campanella that he could so powerfully abstract his attention from fear and the cause of pain as to bear even the rack itself without any severe suffering.

How cruel then to set a patient to watch and weigh and remember their painful sensations. It must not be forgotten that there is indeed a possibility that an invalid may become a prey to pains and sufferings, real pains and sufferings, which have been created almost wholly by the process of long watching and attending to them. I have seen a boy of thirteen who had become so sensitive under this thoughtless regime of practice that he had any symptom belonging to his case which his physician might at the moment speak of in his hearing. This was a case of saint vitus dance. As soon as this boy was put where no one directed his attention to himself and given some mild tonic baths, as soon as he was sent to carry dishes and handle things he must not spill or break, his attention was turned to just the opposite thing, his will was strengthened, and a terrible disease was finally cured.

I saw Mrs. W. and Mrs. B. yesterday.—They have both got bran new babies, and each had her book and her box of little pellets, and every cough or sneeze, every unusual long cry, every time the babies spewed, or looked too ill or too well, they had to take chim. cham. or chin. or nux. rhus. or fus. or bell. caff. or puls. But this is, after all, never-so-much better than that allopathic mother, whose baby arriving about midnight was given whisky before daylight, on the principle, as near as I could make out, that the child ought not to go till morning without taking something, and she did not know what else to give.

